# Characterization and modulation of surface charges to enhance extracellular vesicle isolation in plasma

**DOI:** 10.7150/thno.69094

**Published:** 2022-01-31

**Authors:** Hyun-Kyung Woo, Young Kwan Cho, Chang Yeol Lee, Haeun Lee, Cesar M. Castro, Hakho Lee

**Affiliations:** 1 Center for Systems Biology, Massachusetts General Hospital, Harvard Medical School, Boston, MA 02114, USA.; 2 Department of Radiology, Massachusetts General Hospital, Harvard Medical School, Boston, MA 02114, USA.; 3 Department of Chemistry, Kennedy College of Sciences, University of Massachusetts Lowell, Lowell, Massachusetts, 01854, USA.; 4 Department of Chemistry, Soongsil University, Seoul, 06978, Republic of Korea.; 5 Cancer Center, Massachusetts General Hospital, Harvard Medical School, Boston, MA 02114, USA.

**Keywords:** extracellular vesicles, lipoproteins, surface charge modulation, size-exclusion, cancer

## Abstract

Extracellular vesicles (EVs) carry information inherited from parental cells, having significant potential for disease diagnosis. In blood, however, EVs are outnumbered >10^4^-fold by low density lipoproteins (LDLs), yet similar in size and density. These fundamental disadvantages often cause LDL spillover into EV isolates, thus confounding assay results. We hypothesized that EVs can be further separated from LDLs based on electric charge: EVs and LDLs have different lipid composition, which can lead to differential surface charge densities. To test this hypothesis, we modeled and quantified the surface charge of EVs and LDLs, and used the information to optimally separate EVs from LDLs *via* ion-exchange chromatography.

**Methods:** We built an enhanced dual-mode chromatography (eDMC) device which performed i) size-exclusion to remove particles smaller than EVs and LDLs and ii) cation-exchange in an acidic elution to retain LDLs longer than EVs. The performance of the eDMC, in comparison to size-exclusion only, was evaluated by analyzing the yield and purity of the isolated EVs.

**Results:** By measuring and modeling zeta potentials at different buffer pH, we estimated surface charge densities of EVs (-6.2 mC/m^2^) and LDLs (-3.6 mC/m^2^), revealing that EVs are more negatively charged than LDLs. Furthermore, the charge difference between EVs and LDLs was maximal at a weak acidic condition (pH = 6.4). By applying these findings, we optimized eDMC operation to enrich EVs directly from plasma, depleting >99.8% of LPPs within 30 min. Minimizing LDL contamination improved analytical signals in EV molecular assays, including single vesicle imaging, bulk protein measurements, and mRNA detection.

**Conclusions:** These developments will promote the translational value of the dual-mode separation - a fast, equipment-free, and non-biased way for EV isolation from plasma samples.

## Introduction

Blood contains different types of indigenous nanoscale vesicles that carry clinical information 1, 2. Here, lipoprotein (LPP) particles are most abundant 3, and their amount directly related to heart disease risk 4, 5. Extracellular vesicles (EVs), which are actively shed by cells, contain cellular constituents with strong potential to serve as surrogate disease biomarkers, including cancer 6, neurodegeneration 7, and metabolic disorders 8. Purifying these types of vesicle would help achieve high accuracy in downstream molecular analyses. Such unmet needs are more pronounced in EV assays: EVs are markedly outnumbered by LPPs (>10^5^) 9. A disadvantage that interferes with their analytical measurements (*i.e.*, low signal to background ratio) 10, 11. EVs circulating in blood are conventionally enriched *via* density-gradient ultracentrifugation or size-exclusion chromatography. Both methods, however, suffer from LPP contamination, particularly the spillover of (very) low-density lipoproteins [(V)LDLs], as (V)LDLs and EVs share similar biophysical properties (*e.g.*, density and size; see **Figure S1A**) 11.

We hypothesized that EVs can be further differentiated from LDLs according to electrical properties. Notably, LDLs and EVs have phospholipids yet with varying compositions. LDLs almost exclusively consist of phosphatidylcholine and sphingomyelin 12, both of which are zwitterionic (no net charge) in normal physiological conditions. EVs, on the other hand, have a cell membrane structure (lipid bilayer) 13, containing anionic phosphatidylserine in addition to phosphatidylcholine and sphingomyelin. This compositional dissimilarity would render EVs more negatively charged than LDLs. Importantly, the charge difference could be even larger between cancer-derived EVs and LDLs, as cancer cells exhibit more phosphatidylserine on their outer membrane than normal cells 14, 15. Electric charge can thus be another orthogonal axis to readily distinguish EVs from LDLs (**Figure S1B**).

Here, we present an optimal chromatographic strategy to enrich cancer-derived EVs from blood plasma. As a first step, we quantified EV and LDL surface charges by measuring the zeta potentials of EV or LDL samples across varying pH conditions. Subsequent theoretical modeling estimated differential charge densities at physiological pH (= 7.4), with EVs (-6.2 mC/m^2^) more negatively charged than LDLs (-3.6 mC/m^2^). The analysis also indicated that charge differences between EVs and LDLs were maximized at a weak acidic condition (pH = 6.4). We thus designed an enhanced dual-mode chromatographic approach: i) size exclusion to remove high density lipoproteins (HDLs) that are smaller than EVs and LDLs; and ii) subsequent ion exchange in an acidic condition to separate EVs from LDLs. The method removed >99.8% of LPPs from input blood plasma, and importantly, the acidic elution improved the purity of isolated EVs (>180%) compared to elution in neutral conditions. Consequently, our eDMC processing yielded higher analytical signal for downstream molecular analyses, including single EV protein imaging, bulk protein measurements, and EV mRNA detection.

## Results and Discussion

### Physical properties of LPPs and EVs in human plasma

**Figure 1:**
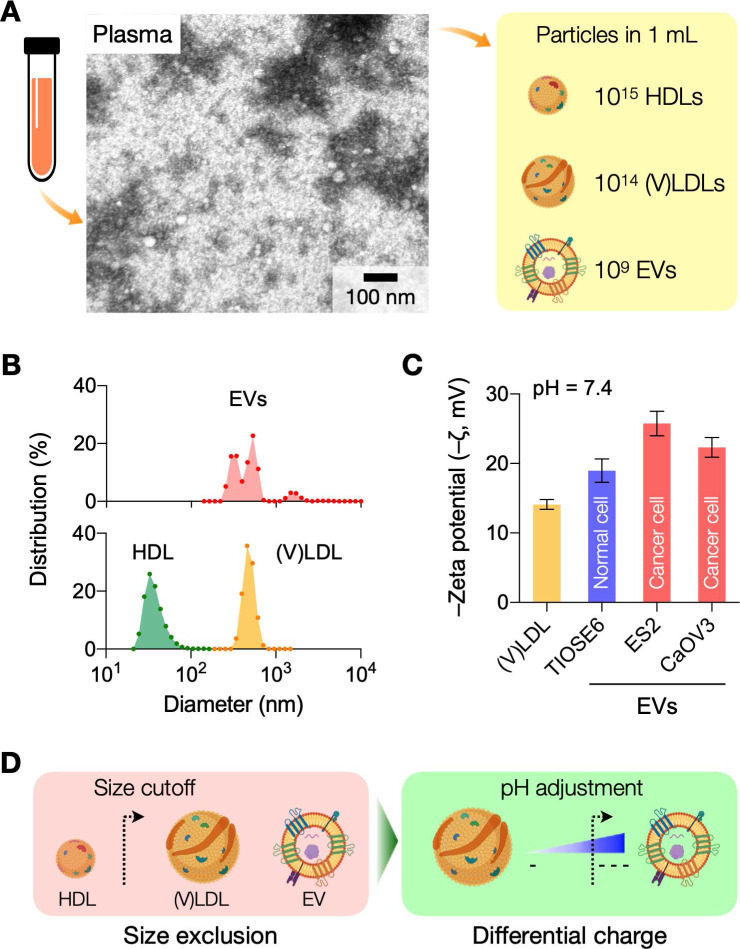
** Rationale and principle of the dual-mode separation. (A)** Representative transmission electron micrograph of a native plasma sample (left). Soluble proteins (white cloud) form broad background. Lipoprotein (LPP) particles, which have a white, circular appearance, dominate the particulate population, with negligible presence of extracellular vesicles (EVs). The observation reflects the substantial imbalance between LPP and EV numbers in plasma (right). HDL, high density lipoprotein; (V)LDL, (very) low density lipoprotein. **(B)** Size comparison between EVs and LPPs. HDLs were smaller (< 100 nm) than EVs and (V)LDLs, while the size of EVs and (V)LDLs overlapped. Hydrodynamic diameter was measured *via* dynamic light scattering. **(C)** Comparison of zeta (ζ) potentials between EVs and (V)LDLs. At physiological pH (7.4), the potential values of (V)LDLs were less negative than EVs derived from normal (TIOSE6) or cancer (ES2, CaOV3) cells. Data from technical duplicates are displayed as mean ± s.d. **(D)** Two biophysical properties are exploited to isolate EVs from plasma. HDLs are first excluded from EVs and (V)LDLs based on size. EVs are subsequently separated from LDLs based on surface charge. Bulk pH is varied to maximize the charge difference.

Transmission electron microscopy identified heterogeneous small particle components in human plasma (**Figure 1A**): abundant soluble proteins formed a cloudy background, and a majority of observed particulates were LPPs (white spherical particles) that outnumbered EVs (see **Figure S2** for images of each particle type). We compared the size distribution of EVs and LPPs using samples of a single-particle type (see Methods for sample preparation). Among EVs and LPPs, high density lipoprotein (HDL) particles could be distinguished for their smaller size (mean hydrodynamic diameter, 38 nm), whereas EVs (484 nm) and (V)LDL (556 nm) had overlapping size distributions (**Figure 1B**). Zeta (ζ) potential measurements, however, revealed that EVs were more negatively charged than (V)LDL particles (**Figure 1C**). Cancer-cell derived EVs had potential values even more negative than benign cell-derived EVs conceivably due to increased exposure of anionic phosphatidylserine on cancer cell membranes 16. Built on these observations, we reasoned a two-step approach to enrich cancer-derived EVs (**Figure 1D**): i) an initial depletion of HDLs through size-based filtering; and ii) follow-up separation of EVs from (V)LDL by exploiting charge differences.

### Characterization of surface charges in LDL and EVs

**Figure 2:**
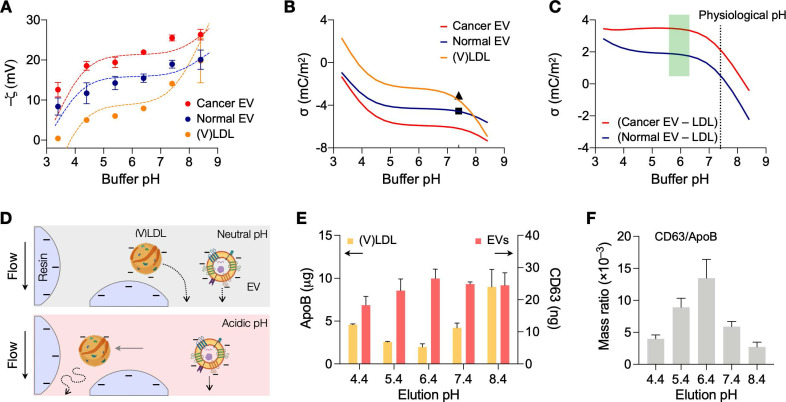
** Analyses of surface charges in (V)LDLs and EVs. (A)** Zeta potentials of (V)LDLs and EVs were measured (solid circles) in buffers at different pH conditions. Cancer EVs displayed more negative potential than (V)LDLs and normal EVs. The measured data were fitted to a theoretical model (dashed lines) that considered pH-dependent ionization of COOH and NH_2_ groups on vesicle surface. Data are displayed as mean ± s.e.m (technical triplicates); some error bars are masked by symbols. **(B)** The net surface charge density (*σ*) estimated from the fitting model was plotted as a function of pH. Cancer EVs were most negatively charged. Symbols indicate *σ* values (pH 7.4) reported in other studies for EVs (◼) 24 and for LDLs (▲) 23. **(C)** The estimated *σ* differences between EVs and (V)LDLs were plotted. The difference would be maximal in a weak acidic condition, pH = 6.4. **(D)** Differential retention of charged vesicles in ion exchange columns. (V)LDLs were less negatively charged than EVs, thereby slower in passing through a cation exchange column. With the increased charge difference in the acidic pH, (V)LDLs would stay longer in the column. **(E)** Amounts of (V)LDLs (yellow bars) and EVs (red bars) eluted from a cation exchange column. At the elution pH of 6.4, the amount was the least for (V)LDLs (i.e., most depletion) and the largest for EVs (highest recovery). Data from technical duplicates are displayed as mean ± s.d. EVs from CaOV3 cells were used. **(F)** The purity of an EV sample was defined as the mass ratio between CD63 and ApoB. The highest purity was observed at the elution pH of 6.4.

We first determined optimal conditions for charge-based separation by characterizing the surface charges of LDLs and EVs. Samples were prepared to contain a single vesicle type (see Methods for details). (V)LDLs were isolated from benign human plasma. EVs were collected from cell-culture supernatant of ovarian cancer cells (CaOV3) and nonmalignant ovarian epithelium cells (TIOSE6); using cell culture ensured the collection of pure EVs in sufficient amounts (>10^9^ EVs/mL) for reliable measurements of zeta potential. Isolated vesicles were then spiked in buffers, and ζ potentials were measured at different bulk pH (3.4 - 8.4) conditions. Over the pH range tested, EVs had more negative ζ values than (V)LDL; among EV types, cancer-derived EVs showed lower ζ values than normal EVs (**Figure 2A**).

We further analyzed the data by applying the site-dissociation model that relates the surface electrical potential with pH 17. Assuming that changes comes from ionization of acidic (-COOH) and basic (-NH_2_) residues on the vesicle surface, the charge density (*σ*) is estimated as,


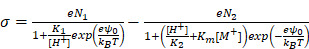

(1)

where *N*_1_ and *N*_2_ are the surface densities of NH_2_ and COOH groups, respectively; *K*_1_ and *K*_2_ are the acid dissociation constants for the respective groups; *e* is the electron charge; *k_B_* is the Boltzmann constant; *T* is the solution temperature; and *ψ*_0_ is the potential at the lipid surface 18, 19. The effect of bulk pH and electrolyte is taken into account by including proton (H^+^) and cation (M^+^) concentrations (in molarity). The binding constant *K_m_* reflects the binding of cation to the ionized acidic residue 20. From an electrokinetic model (Gouy-Chapman formula), we obtain another relationship linking *ψ*_0_ and *σ,*


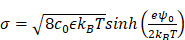

(2)

where *c*_0_ is the concentration of the electrolyte, and *ε* is the permittivity of water. Finally, the ζ potential, which is measured at the hydrodynamic shear plane, can be related to *ψ*_0_ by solving the Poisson-Boltzmann equation,


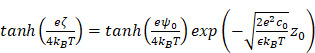

(3)

where *z*_0_ is the distance between the shear plane and the lipid surface.

From Eq. (3) and measured ζ values, we calculated *ψ*_0_, setting *z*_0_ = 0.2 nm for phospholipids 21. We then used Eqs. (1) and (2) to find *N*_1_ and *N*_2_ that best fit the measured data. Dashed lines in **Figure 2A** showed calculated ζ as a function of bulk pH. Using the fitting model, we could estimate the net surface charge density *σ* (**Figure 2B**). Overall, EVs had more negative charges than LDLs, and both particle types had charge values decreasing in basic buffer condition. This observation also matched with differential movements of EVs and LDLs seen in electrophoretic measurements 22. At the physiological pH (7.4), the computed *σ* values were -3.6 mC/m^2^ for (V)LDLs and -6.2 mC/m^2^ for cancer EVs, whose values agreed with previous reports 23, 24. We also found that a slightly acidic pH maximizes the charge difference between EVs and (V)LDL (**Figure 2C**).

### Differential vesicle filtering based on charge gradient

Surface charge differences can affect the retention time of vesicles during cation-exchange chromatography (**Figure 2D**): less negatively charged vesicles would exit later, as they are more effectively trapped in the filtering matrix. To test the reasoning, we first used isogenic EVs with different surface charges. EVs from cell culture (CaOV3) were biotinylated and labeled with streptavidin (see Methods for details); this labeling made EVs less negatively charged, increasing ζ values from -21.5 to -6.3 mV (**Figure S3A**). When unaltered and labeled EV samples were filtered *via* cation-exchange chromatography, labeled EVs (less negatively charged) were collected later than unaltered ones (**Figure S3B**).

We next compared the differential retention between (V)LDLs and EVs. We processed human plasma samples, spiked with CaOV3 EVs, through cation-change columns. The pH of elution buffer was varied to modulate surface charge differences between (V)LDLs and EVs. **Figure 2E** shows the amount of (V)LDLs and CD63 in the EV elution fraction. The amount of (V)LDLs was the lowest and CD63 the largest at pH = 6.4, which agreed with the maximum charge difference between these two vesicle types. Other tetraspanins (CD9, CD81) associated with EVs also showed elution patterns similar to that of CD63 (**Figure S4**). At low pH, we observed the increase of LDLs in the elution fraction. This could be attributed to the competition between highly protonated EVs and LDLs in the cation-exchange column, which led to inefficient LDL retention by the column.

As a single quality metric of EV preparation 10, we also calculated the EV purity by taking the mass ratio of CD63 and ApoB in a given sample (**Figure 2F**), which would better reflect EV purity with molecular specificity 25. Eluting samples in weak acidic conditions yielded EV purity higher than processing at physiological pH of 7.4.

### Single column device for EV isolation

**Figure 3:**
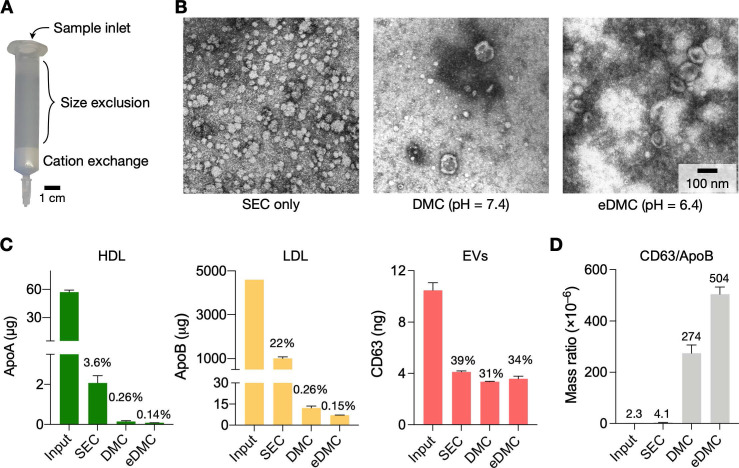
** Characterization of EV isolation methods. (A)** Design of the dual-mode chromatography (DMC) column. The column has two separation layers, one for size exclusion (top) and the other for cation exchange (bottom). The small analytes such as HDLs and soluble proteins are retained in the top layer. The bottom layer preferentially holds less negatively charged particles such as (V)LDLs. **(B)** Transmission electron micrographs of plasma samples after different chromatographic separations: SE chromatography (SEC), DMC (pH = 7.4), and enhanced DMC (eDMC, pH = 6.4). LPPs (white particles) were notably removed after DMC operation, and more EVs were seen in the eDMC-processed sample. **(C)** The amounts of LPPs and EVs were quantified before and after chromatographic separations. Overall, eDMC showed the highest depletion of both HDLs and (V)LDLs. EV recovery rate was similar between three chromatographic methods. Data are displayed as mean ± s.d. from technical duplicates. **(D)** The sample purity (mass ratio between CD63 and ApoB) was compared. Note that eDMC increased the purity >120-fold compared to the SEC-only separation. Data from technical duplicates are displayed as mean ± s.d.

We next set up the entire process for EV isolation from plasma. Two filtering steps were necessary: size exclusion chromatography (SEC) and cation exchange (CE) to remove HDLs and (V)LDLs, respectively. We constructed a dual-mode chromatography (DMC) column that performed both SEC and CE filtering from single sample loading (**Figure 3A**; see Methods for details) 25. The combined device not only simplified the EV isolation, but also produced higher EV purity than the separate operation of SEC and CE (**Figure S5**). The input sample volume was set to 0.5 mL of plasma.

**Figure 3B** shows electron micrographs of plasma samples following different filtering processes. Using SEC alone, we noticed a large number of LPPs, mostly (V)LDLs, remaining in the filtered plasma (**Figure 3B**, left). Adding the CE step in tandem (i.e., DMC) markedly reduced LPPs (**Figure 3B**, middle), and the acidic elution (eDMC) led to higher EV purity (**Figure 3B**, right).

Analytical measurements confirmed these observations. We measured the amount of total protein, HDL, (V)LDL, and CD63 before and after filtration. All filtration methods removed most (>98%) of total protein (**Figure S6A**), but their efficiency was different in removing LPPs (**Figure 3C**). SEC alone effectively removed HDLs (>97% of the initial load) from plasma, but not (V)LDLs (22% of initial loading remained). With DMC (pH = 7.4), the residual LPP contents decreased to 0.26% of initial loading, which was further lowered to 0.15% with eDMC (pH = 6.4). EV contents in the eluate, estimated by CD63 amounts, also improved at the weak acidic condition. Importantly, the eDMC processing improved EV purity by >120-fold when compared to SEC processing alone (**Figure 3D; Figure S6B**). We also compared SEC, DMC, and eDMC performance with urine samples. Unlike plasma, the amount of urinary LPPs was low, and all three methods produced similar results for LPPs and EVs (**Figure S7**).

We further increased the input plasma volume (1 and 2 mL) and processed them *via* eDMC. With 2-mL plasma samples, LDL contamination increased in the collected EV fraction, which in turn deteriorated EV purity (**Figure S8**). Considering these factors, we set the recommended plasma volume to ≤1 mL. The processing time of 1 mL plasma was about 30 min.

### Plasma EV analyses

**Figure 4:**
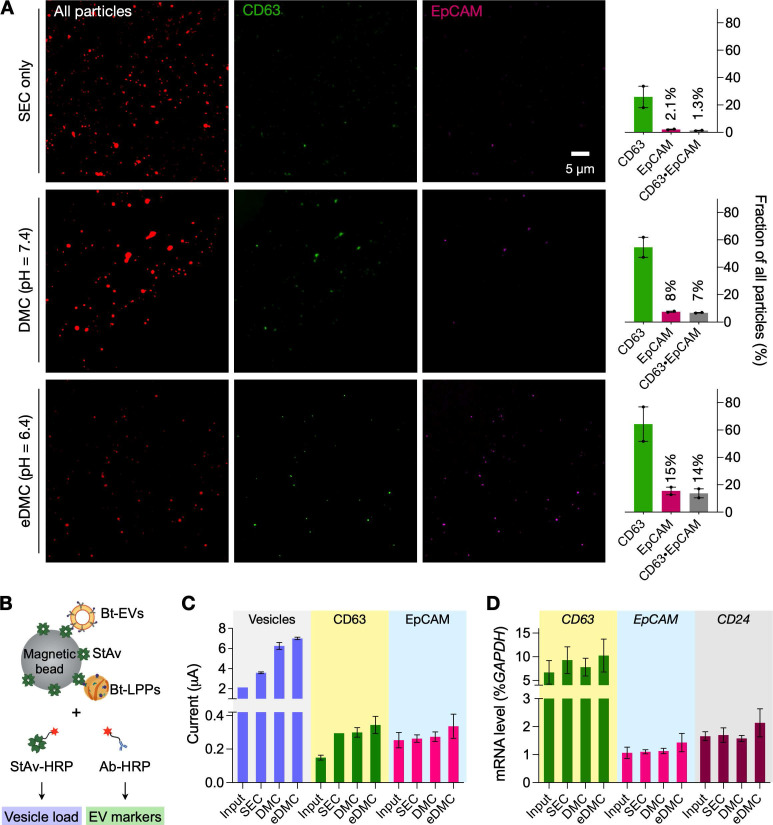
** Downstream molecular analyses of purified plasma samples. (A)** Single particle imaging. Human plasma samples spiked with cancer EVs (CaOV3) were processed by SEC (top row), DMC (middle) and eDMC (bottom row). Isolated particles were labeled for protein (red), CD63 (green), and EpCAM (magenta). The total number of particles was larger with SEC than DMC or eDMC processing. The fraction of particles showing EV-associated markers (CD63, EpCAM), however, were highest in the eDMC-processed sample (bar graphs), indicating the most efficient EV enrichment. Bars show mean ± s.d from technical duplicates (*n* = 2). **(B)** Integrated magneto-electrochemical exosome (iMEX) assay was used for bulk protein analyses. Particles in SEC, DMC and eDMC eluate were biotinylated (Bt), captured on streptavidin (StAv)-coated magnetic beads, and then labeled with detection probes: StAv to estimate total vesicle load or antibody (Ab) against target EV proteins. All detection probes were conjugated with horseradish peroxidase (HRP) to generate electrical current in the presence of 3,3',5,5'-tetramethylbenzidine. **(C)** iMEX results for vesicle load, CD63, and EpCAM. Native plasma showed the lowest signal for all markers, presumably because vesicle capture was hindered by nonspecific protein adsorption on beads. The eDMC-processing produced the highest analytical signal. Data from technical duplicates (*n* = 2) are displayed as mean ± s.d. **(D)** mRNA analyses of native, SEC-, DMC- and eDMC-processed plasma samples. EV-associated markers were the highest in the eDMC processed samples. Data are displayed as as mean ± s.d (*n* = 3).

We examined the effect of different EV preparations on downstream molecular analyses. We prepared test samples by spiking cancer-derived EVs (6 × 10^10^ EVs) into healthy donors' plasma and filtered aliquots *via* SEC, DMC (pH = 7.4), and eDMC (pH = 6.4) columns for comparison. We first assessed the purity of filtered samples through single-particle imaging (see Methods for details) 26, 27. To universally identify lipid particles, we incubated eluate with amine-reactive dyes (Alexa Fluor 555 succinimidyl ester) that can label surface proteins. Labeled particles were then captured on a glass substrate and further stained for CD63 (EV marker) and EpCAM (cancer marker) immunofluorescence. Fluorescent microscopy confirmed superior EV-enrichment *via* eDMC processing (**Figure 4A**). Although the SEC-only sample had more lipid particles than DMC- and eDMC-processes sample, the fraction of CD63-positive signal accounted for ~26% of all particles while EpCAM-positive signal was only in 2.1%. CD63-positive and EpCAM positive fractions increased when dual-mode columns were used, with the eDMC-processed samples having the highest number for both fractions (64% for CD63-positive, and 15% for EpCAM-positive).

We next performed bulk assays for protein and mRNA targets. For protein measurements, we used the established integrated magneto-electrochemical exosome (iMEX) platform (see Methods for details) 28-30. We biotinylated lipid vesicles with amine-reactive biotin and captured them on streptavidin-coated magnetic beads. Analytical signal (electrical current) was generated by labeling captured vesicles with an oxidizing enzyme (horseradish peroxidase, HRP) and mixing the conjugates with chromogenic electron mediators (**Figure 4B**). We used streptavidin-HRP as a labeling probe to estimate total vesicle amount; and antibody-HRP to detect target proteins (CD63, EpCAM). The iMEX assay results are shown in **Figure 4C**. Unfiltered plasma yielded the lowest signal, presumably due to fouling effects: abundant plasma proteins nonspecifically adsorbed on beads to hinder vesicle capture. Purifying vesicles increased the analytical signal, with eDMC filtering showing overall higher values.

Bulk mRNA analyses produced similar results (**Figure 4D**). We isolated total RNA from native and filtered (SEC or eDMC) samples, and analyzed them for expression of *GAPDH* (loading control), *CD63* (EV-enriched), *EpCAM* (ovarian cancer) and *CD24* (ovarian cancer). All three types of samples (native plasma, SEC filtered, eDMC filtered) showed similar GAPDH expressions (**Figure S9**), while eDMC filtering produced the highest expression of EV (*CD63*) and cancer associated markers (*EpCAM and CD24*).

**Figure 5:**
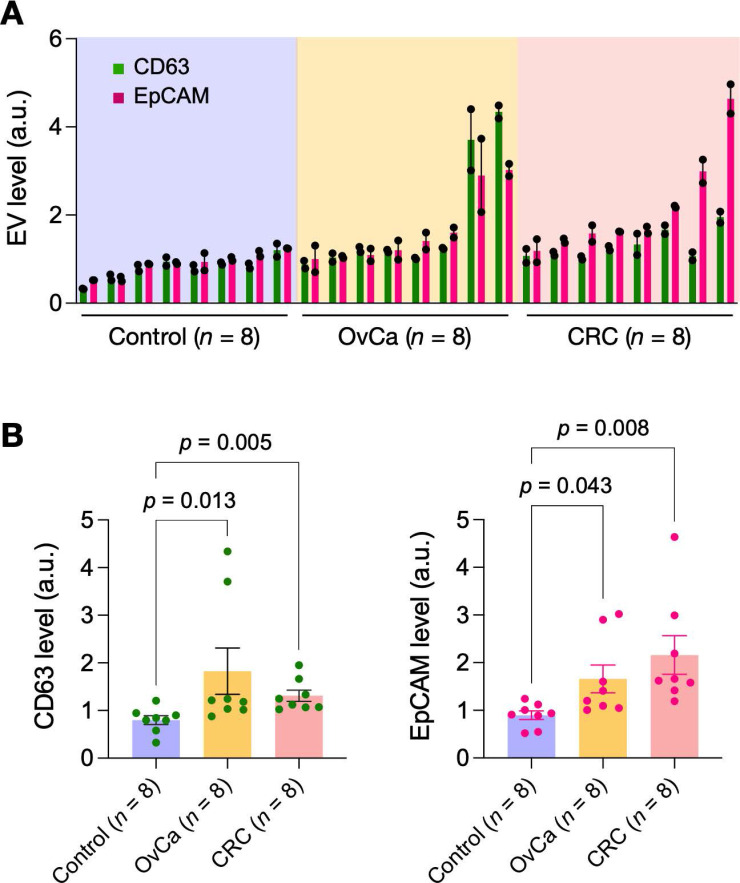
** Pilot testing with clinical samples.** Plasma samples from healthy donors (*n* = 8), ovarian cancer patients (*n* = 8) and colorectal cancer patients (*n* = 8) were processed by eDMC and then analyzed by iMEX for CD63 and EpCAM expression. Data from technical duplicates are displayed as mean ± s.d. OvCa, ovarian cancer; CRC, colorectal cancer. **(B)** CD63 and EpCAM expressions were higher in cancer patient plasma. Dunn's multiple comparisons test was used.

Lastly, we applied eDMC to process clinical samples for iMEX measurements. EVs were isolated from plasma samples of healthy donors (*n* = 4), ovarian-cancer patients (*n* = 4), and colorectal cancer patients (*n* = 4). Isolated EVs were then captured on magnetic beads and further labeled for CD63 and EpCAM. Control samples were labeled with isotype-matched IgG. Samples from cancer patients displayed higher expression of CD63 and EpCAM than non-cancer controls (**Figure 5**), consistent with prior findings 28, 30, 31. In contrast, particle concentrations (measured *via* nanoparticle tracking analysis) showed no significant difference between healthy and cancer cohorts (**Figure S10**); this artifact could be caused by residual (V)LDLs present in eDMC filtrates.

## Conclusions

EVs and LPPs' overlapping physical properties often challenge EV-focused investigation. Conventional purification methods (*e.g.*, density-gradient centrifugation, size exclusion, filtration) that rely on one physical parameter, isolate both particle types. In this work, we exploited electrical charges as an additional dimension to set EVs apart from LPPs. Due to differences in phospholipid compositions, EVs have more negative charges than LPPs, a hypothesis that was validated through experimental measurements and theoretical modeling. We further found that the charge difference between EVs and LPPs is maximized at slightly acidic pH (= 6.4). We then exploited two physical properties, size and charge, to differentially elute EVs, LDLs, and HDLs *via* chromatographic separation. The approach enriched an EV population with >99.8% of plasma LLPs removed. In downstream EV protein and mRNA analyses, such high-purity EVs yielded better analytical signals than LPP-contaminated samples. eDMC would be applicable for a broad range of EV populations when the following two conditions are met: i) EV size is greater than the cut-off for size exclusion (~40 nm) 32; and ii) EVs are more negatively charged than LDLs.

We envision the following future directions to expand the scope of the current work. First, an immediate task would be challenging eDMC with various clinical plasma samples to fully assess eDMC's analytical capacity (*e.g.*, saturation concentration, scale-up capacity). In addition, we can further improve the technical aspects of the system. After one-time sample loading, the single column device (**Figure 3A**) requires intermittent buffer injections for elution. Automating this process should enhance throughput and reproducibility of column operations. Second, modifying the current method could improve EV recovery and purity. For example, we could target EVs with antibodies before eDMC; this process will make EVs more negatively charged, thereby concentrating them in earlier elution fractions that are well separated from LDLs. We can also consider functionalizing the column-packing material with affinity ligands to enable additional molecular-sieving (*e.g.*, removing antibody aggregates) or immuno-capture (*e.g.*, targeting cell-specific EVs for depletion or enrichment) along with size exclusion and ion exchange. Third, we seek to perform in-depth analyses on particles collected with dual-mode separation. In our pilot data (**Figure 2**), cancer-derived EVs were more negatively charged than EVs from benign cells. Under this condition, our charge-based separation should preferentially collect cancer EVs, which would improve the accuracy of cancer diagnostics through EV profiling. This reasoning, however, should be validated with a larger panel of cell lines (cancer and benign) and eventually clinical samples derived from diverse patient populations and attendant co-morbidities (*e.g.*, hyperlipidemia, diabetes). It would be equally interesting to evaluate whether LPP-elution fractions contain EV-associated molecular markers as previously reported 33, to rule out EV contamination in LPP samples. These developments will promote the translational value of the dual-mode separation - a fast, equipment-free, and non-biased way for EV isolation from plasma samples.

## Materials and Methods

### Cell lines and culture for EV production

Cells lines used in this study (CaOV3, ES2, and TIOSE6) were purchased from American Type Culture Collection. Cells were seeded in T175 flask and cultured in vendor-recommended media supplemented with 1% exo-free fetal bovine serum (Thermofisher): Dulbecco's modified Eagle medium (Thermofisher) for CaOV3; McCoy's 5A medium (Thermofisher) for ES2; and RPMI medium (Thermofisher) for TIOSE6. For EV collection, cell-culture supernatants (100 µL) were collected and centrifuged at 300 × *g* for 10 min. Following the centrifugation, supernatants were collected and centrifuged again at 2,000 × *g* for 10 min. Finally, clear supernatants were filtered through 0.22-*μ*m membrane filters (cat#430767, Corning), and the filtrates were concentrated using centrifugal filter units (10 kDa cutoff; Centricon-70, Millipore Sigma).

### Human samples

The study protocol was reviewed and approved by the Institutional Review Board of Massachusetts General Hospital (IRB number, 2019P003472). Informed consents were obtained from all participants of this clinical study. Plasma samples were centrifuged at 2,000 × *g* for 3 min to remove cell debris, and supernatants were collected. Five hundred microliters of supernatants diluted in 500 μL of PBS were used for isolation experiments. Urine samples were centrifuged at 500 × g for 10 min, and supernatants were collected and centrifuged again at 2,500 × *g* for 15 min. The supernatants were then concentrated using centrifugal filter units (Centricon-70, 10 kDa cutoff). One milliliter of concentrated urine samples (from 10 mL of urine sample) were used for isolation experiments.

### Lipoprotein purification

Low and very low-density lipoproteins were isolated using LDL/VLDL/HDL purification kits (Cell Biolabs) according to manufacturer protocol. The isolated (V)LDLs were diluted in filtered (0.22 µm) phosphate-buffered saline (fPBS) and used in the subsequent assays. Protein concentrations were determined by Qubit protein assay kits (Thermofisher). A (V)LDL sample (2 µL) was mixed with freshly-prepared Qubit working solution (198 µL) in an assay tube (cat#Q32856, Life Technologies). After 15 min incubation at room temperature (RT), fluorescence intensity was measured using Qubit Fluorometer. The protein concentration was determined based on a standard curve (**Figure S11**).

### Construction of separation columns

Sepharose CL-4B (GE Healthcare) and Fractogel EMD SO_3_^-^ (M) (Millipore Sigma) resins were used after double washing with PBS. A nylon net filter with 11 μm pore size (NY1102500, Millipore Sigma) was cut and placed on the bottom of a 10 mL syringe (BD Biosciences). For the SE-only column, 10 mL of washed Sepharose matrix was stacked on the syringe. In case of the dual-mode column, 2 mL of washed Fractogel resin was loaded on the syringe first, and then 10 mL of washed Sepharose gel was stacked on top. Prepared columns were allowed to settle at least for 24 hours and stored at 4 °C until use.

### Column operation

All columns were washed with 10 mL of fPBS before sample loading. An input sample (1 mL) was introduced into the column, and eluate was continuously collected with addition of fPBS. The pH of fPBS was varied depending on experiments, for example, pH = 7.4 for the SE-only filtering and pH = 6.4 for the enhanced dual-mode filtering. We adjusted the pH of elution buffer by adding HCl (1 M) or NaOH (10 M). The estimated changes in the ionic strength was <1% compared to a neutral buffer (pH = 7.4). Two milliliters of EV-containing eluate fractions were collected after discarding the void volume (non-EV fractions): 3 mL for the SE-only column and 3.5 mL for the dual-mode column. The collected EV samples were concentrated to 100 µL using Exodisc (EX-D1001, LabSpinner) 34. At this time, the buffer was exchanged by adding fPBS (200 μL, pH = 7.4). Downstream assays used samples in 100 μL fPBS (pH = 7.4).

### Nanoparticle tracking analysis (NTA)

NanoSight LM10 (Malvern) equipped with a 405 nm laser was used. Samples were diluted in fPBS to obtain the recommended particle concentration (25-100 particles/frame). For each test sample, three 30-sec videos were recorded (camera level, 14). Recorded videos were analyzed by NTA software (version 3.2) at a detection threshold of 3.

### Zeta potential measurements

Samples were diluted in fPBS (1 mL) with the pH varying from 3.4 to 8.4. The total particle concentration was adjusted to be about 10^9^ particles/mL. The zeta potential was measured by Zetasizer Nano-ZS (Malvern) on technical triplicate samples.

### Enzyme-linked immunosorbent assay (ELISA)

For HDL and (V)LDL quantification, Human ApoA-I and ApoB Duplex ELISA kit (Cell Biolabs) was used according to manufacturer instructions. Samples and standards were measured in duplicate. For CD63 assay, anti-CD63 antibodies (4 μg/mL; see Table S1 for antibody information) were loaded (50 μL/well) in a 96 well-plate (Nunc MaxiSorp flat-bottom, Thermofisher) and incubated overnight at 4 °C. After washing twice with 200 μL of 0.1% BSA in PBS, plates were blocked with 1% BSA (200 μL/well) for 2 h at RT. After washing twice as described before, biotinylated anti-CD63 antibodies (500 ng/mL) were loaded (50 *μ*L/well) and incubated for 1 h at RT. After washing twice, HRP-conjugated streptavidin (1:20,000 diluted in 0.1% BSA; #405210, BioLegend) were loaded (50 μL/well) and incubated for 20 min at RT. After washing three times, 100 μL of 3,3',5,5'-tetramethylbenzidine (TMB, BioLegend) were loaded per well and incubated for 30 min at RT. Reactions were stopped by adding 50 μL of stop solution, and absorbance was measured at 450 nm on a plate reader (Tecan). To estimate CD63-positive EV numbers, a calibration curve was generated using serially diluted EV samples whose EV numbers were counted by NTA (**Figure S10**).

### Single EV imaging

Three microliters of input samples were mixed with 2 μL of 0.1M Na_2_CO_3_ buffer, and the mixture was incubated with 0.2 μL of Alexa Fluor 555 NHS ester (10 mg/mL in DMSO; #A37571, Thermofisher Scientific) for 1 h at RT. Excess dyes were removed *via* washing with Zeba micro spin desalting columns (#87765, Thermofisher Scientific). After two-time washing, 3 μL of dye-labeled samples were loaded on a glass slide and allowed to settle (30 min, RT). The slide was then washed with fPBS and incubated with 10 μL of fixation buffer (4% paraformaldehyde) and washed. For CD63 detection, captured vesicles were labeled (90 min, RT) with biotinylated anti-CD63 antibody (10 μg/mL), washed in fPBS, and incubated (30 min, RT) with FITC-streptavidin secondary reagents (5 μg/mL). For EpCAM detection, vesicles were incubated (90 min, RT) with anti-EpCAM antibody (10 μg/mL), washed in fPBS, and incubated (30 min, RT) with Alexa Fluor 647 anti-mouse antibody (5 μg/mL). After the final washing step, fluorescence images were taken by an inverted microscope (Nikon, Eclipse TE2000S) equipped with an sCMOS camera (Andor, Zyla). Image analyses were performed using ImageJ.

### Integrated magneto-electrochemical exosome (iMEX) assay

The detailed assay process is described in previous studies 28-30. In brief, 100 μL of streptavidin-coated magnetic beads (Dynabeads M-280 streptavidin;#11205D) were washed in PBS (1 mL) for 10 min at RT. After washing, beads were re-suspended in 50 μL of superblock buffer (#37580, Thermofisher) for 3 h at RT. Input samples were biotinylated by incubating them with 1 μL of 10 mM biotin EZ-Link Sulfo-NHS-LC-Biotin (Thermofisher scientific) for 30 min at RT. Excess biotin was removed by two-time washing in Exodisc with the injection of 500 μL of PBS 34. Biotinylated samples were incubated (1 h, RT) with 1:1 (v/v) mixture of HAMA blocker (#ab193969, Abcam) and superblock buffer (Thermofisher). For the iMEX assay, 80 μL of biotinylated samples were mixed with 2.5 μL of the magnetic bead solution (30 min at RT). After incubation, magnetic beads were washed with 30 μL of PBS containing 0.05% (v/v) Tween 20. Then, the beads were mixed with either 30 μL of antibodies (4 μg/mL in 0.1% BSA) conjugated with horseradish peroxidase (HRP) or 30 µL of streptavidin-HRP (1:1000 in 0.1% BSA, #405210). The mixture was incubated for 15 min at RT. Finally, beads were washed twice with 30 μL of PBS. Seven microliter of the prepared beads solution and 30 μL of TMB solution (BioLegend) were loaded on top of an electrode. After 3 min, chronoamperometry measurement was performed. The measured current values between 45 and 50 sec were averaged.

### RNA analyses

Total RNA from prepared samples was extracted using miRNeasy kit (Qiagen). Extracted RNA was then added to cDNA synthesis mix (SuperScriptTM VILOTM cDNA Synthesis Kit, Invitrogen) composed of 1× SuperScriptTM Enzyme Mix and 1× VILO™ Reaction Mix. cDNA was prepared with the following incubation protocol: 25 °C, 10 min; 42 °C, 60 min; 80 °C, 5 min for enzyme inactivation. As-prepared cDNA was added to qRT-PCR reaction mix composed of 1× TaqManTM Fast Advanced Master Mix (ThermoFisher Scientific) and 1× gene-specific TaqManTM Gene Expression Assay Mix (ThermoFisher Scientific). qRT-PCR was conducted on CFX Opus 96 real-time PCR instrument (Bio-Rad) with the following steps: 95 °C, 1 min and then subsequent thermal cycling schedule (55 cycles): 95 °C, 3 sec; 60 °C, 30 sec; fluorescence measurement. Setting the fluorescent threshold value to 400, quantification cycle (*C_q_*) values were determined by the system software.

## Supplementary Material

Supplementary figures, table, and note.
