# Supplementary material for: Characterization and modulation of surface charges to enhance extracellular vesicle isolation in plasma
Source: Theranostics. 2022 Jan 31;12(5):1988–98. doi: 10.7150/thno.69094 (PMC8899565; doi:10.7150/thno.69094)
Supplement: Supplementary file 1 — Supplementary figures, table, and note. [file thnov12p1988s1.pdf]

SUPPLEMENTARY INFORMATION

Characterization and modulation of surface charges to enhance extracellular vesicle isolation in plasma

Hyun-Kyung Woo[1,2], Young Kwan Cho[1,3], Chang Yeol Lee[1,2], Haeun Lee[1,4], Cesar M. Castro[1,5], Hakho Lee[1,2]*

1. Center for Systems Biology, Massachusetts General Hospital, Harvard Medical School, Boston, MA 02114, USA
2. Department of Radiology, Massachusetts General Hospital, Harvard Medical School, Boston, MA 02114, USA
3. Department of Chemistry, Kennedy College of Sciences, University of Massachusetts Lowell, Lowell, Massachusetts, 01854, USA
4. Department of Chemistry, Soongsil University, Seoul, 06978, Republic of Korea
5. Cancer Center, Massachusetts General Hospital, Harvard Medical School, Boston, MA 02114, USA

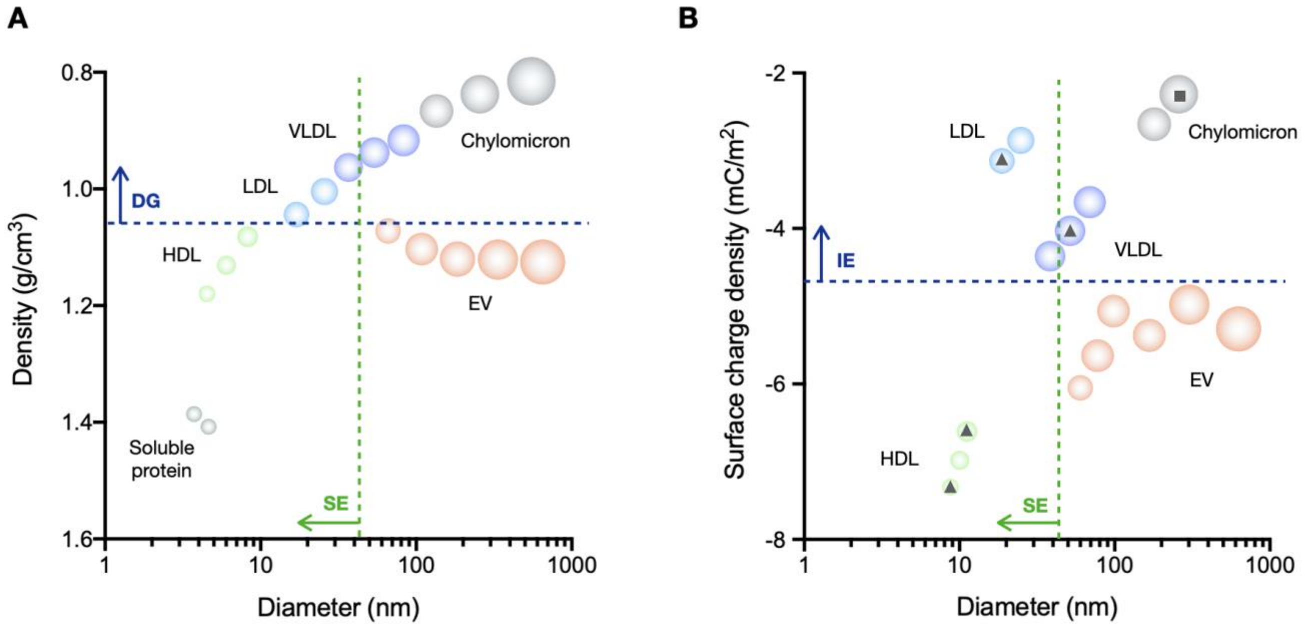

Figure S1. Schematic overview of size distribution for LPPs and EVs. (A) Size and density distribution represent the overlap between LDL (blue) and extracellular vesicles (orange). EVs are separated by size exclusion (SE) and density gradient (DG) from LPPs. (B) Size and surface charge density distribution represent another orthogonal axis (SE and ion exchange (IE)) to distinguish between LDL and EVs. Symbols indicate surface charge density values reported in other studies for (V)LDL and HDL (▲) [1], and large size of LPPs (chylomicron; ■) [2].

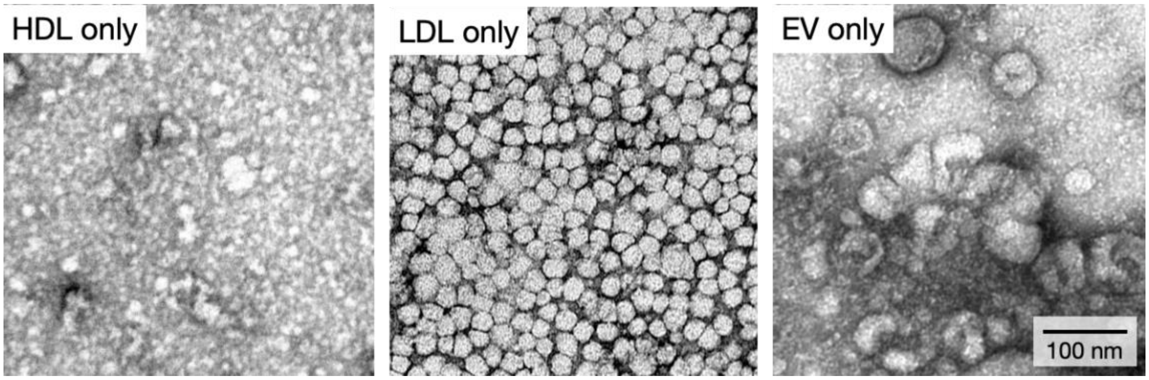

Figure S2. Transmission electron micrographs of HDL-, LDL-, and EV-only particles. HDL and LDL particles were isolated from plasma; EVs were from cell culture.

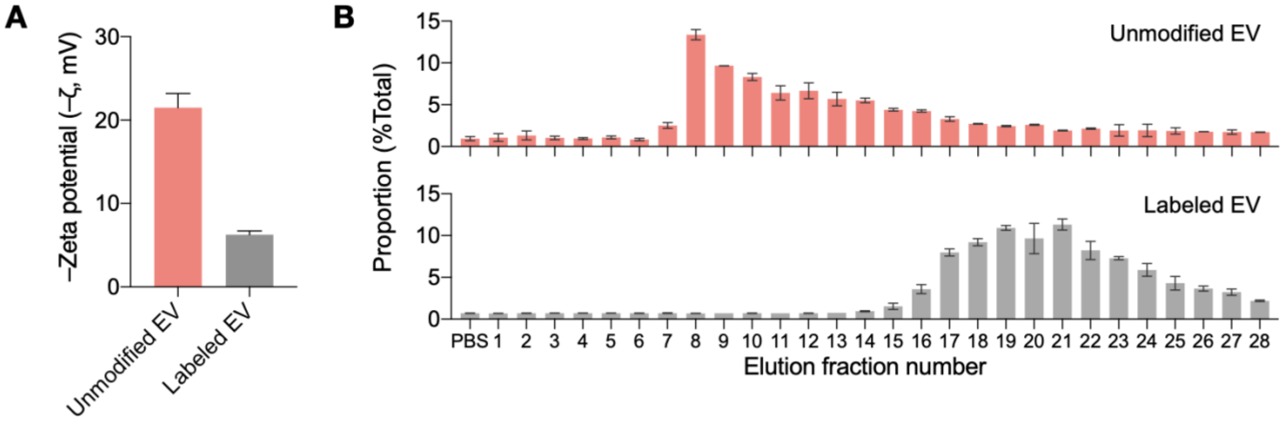

Figure S3. Differential elution of EVs based on their surface charge. **(A)** EVs from CaOV3 cells were biotinylated and labeled with streptavidin. At pH 6.4, labeled EVs showed less negative zeta potential than unmodified EVs from the same cell line. **(B)** Both EV samples were processed by cation exchange chromatography, and eluate sample fractions were analyzed. Note that labeled EVs (less-negatively charged) eluted later than unmodified EVs (more negatively charged). Data are displayed as mean ± s.d from technical duplicates.

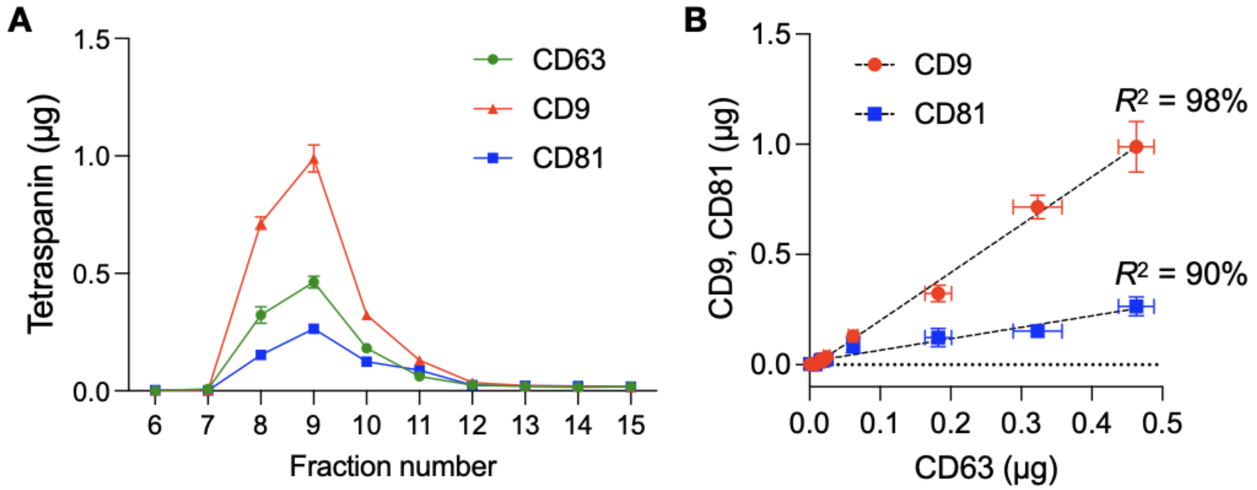

Figure S4. Quantification of tetraspanins in different elution fraction. (A) Plasma samples (0.5 mL) were processed by eDMC, and the amount of CD63, CD9, and CD81 was measured in different elution fractions. Similar elution patterns among markers were observed. **(B)** The amount of CD9 and CD81 in a given elution fraction was linearly correlated with that of CD63. Data are displayed as mean ± s.d from quadruplicate measurements.

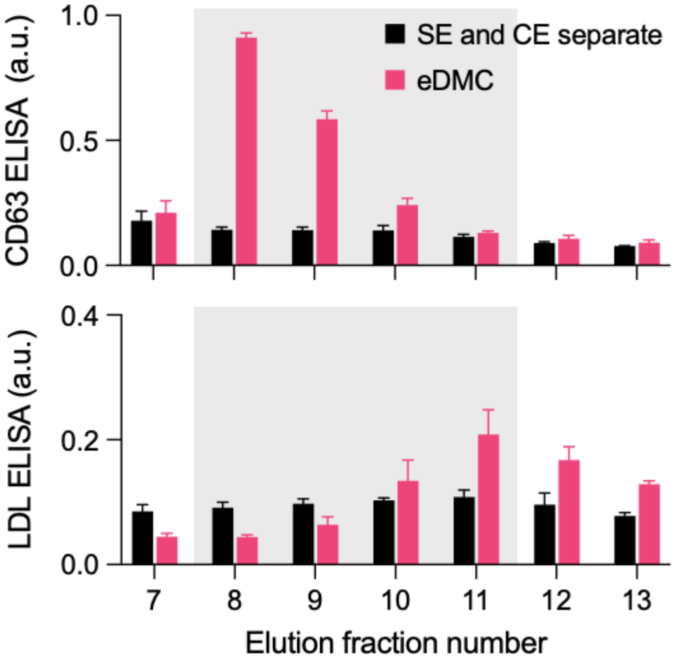

Figure S5. Comparison of separate and combined processes of size exclusion (SE) and cation exchange (CE) chromatography. In the same void fraction and void fraction, combined process resulted in higher CD63 signal and lower LDL contamination. The grey shadow indicates EV-elution fractions. Data are displayed as mean ± s.d from duplicate measurements.

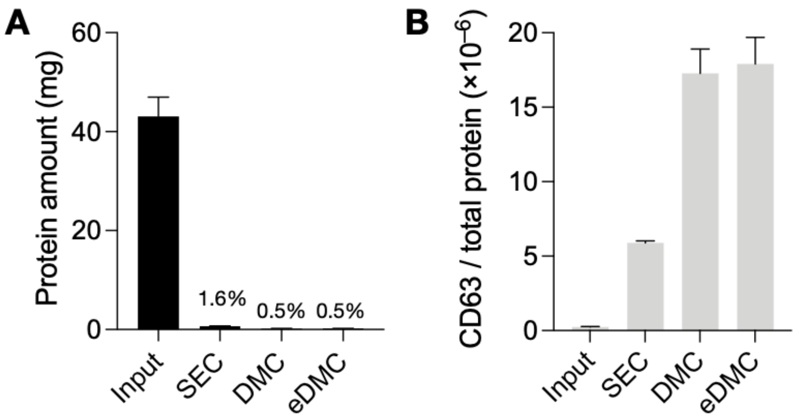

Figure S6. **EV purity against total protein. (A)** Total protein amount (0.5 mL plasma) was measured and compared between three chromatographic methods. Both DMC and eDMC removed >99% of total proteins. **(B)** The mass ratio of CD63 to total protein was compared. eDMC filtrate had the highest purity.

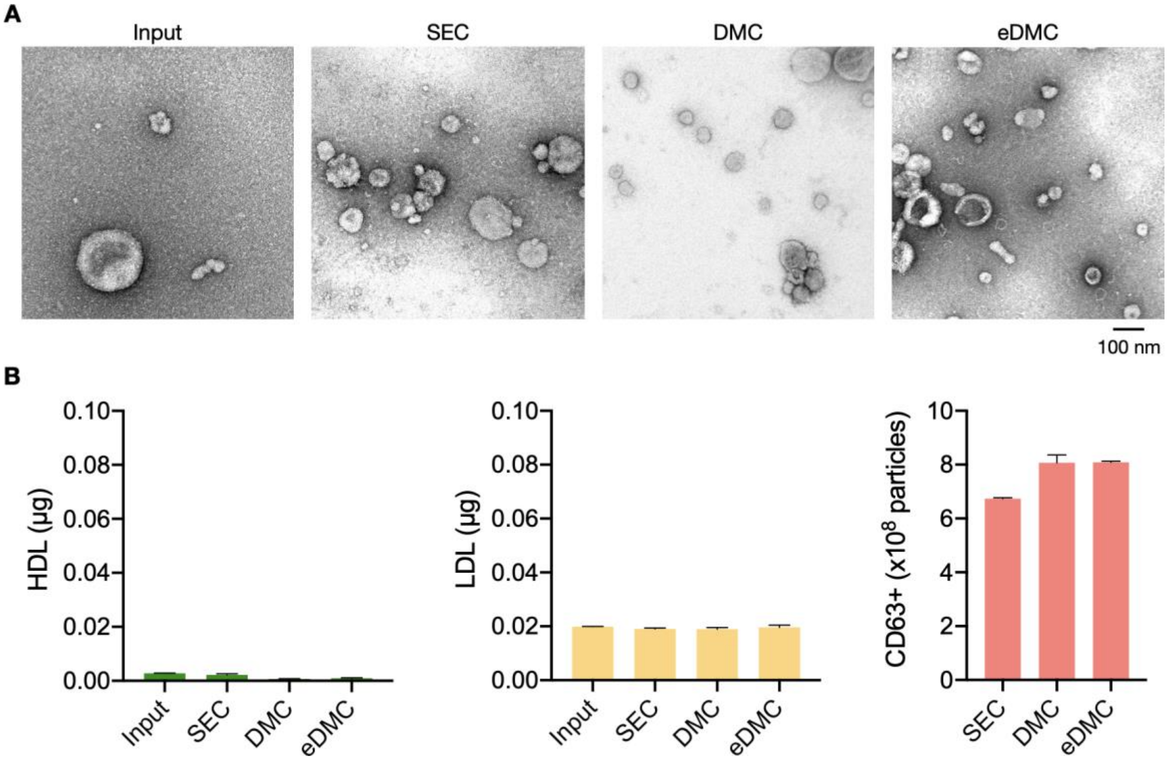

Figure S7. Urine sample processing. Urine samples spiked with EVs were used for particle isolation. **(A)** Transmission electron micrographs for input sample and EVs isolated by using SEC, DMC and eDMC. **(B)** After isolating EVs using three different methods, recovery of particles were estimated by ELISA. The technical duplicates are displayed as mean ± s.d.

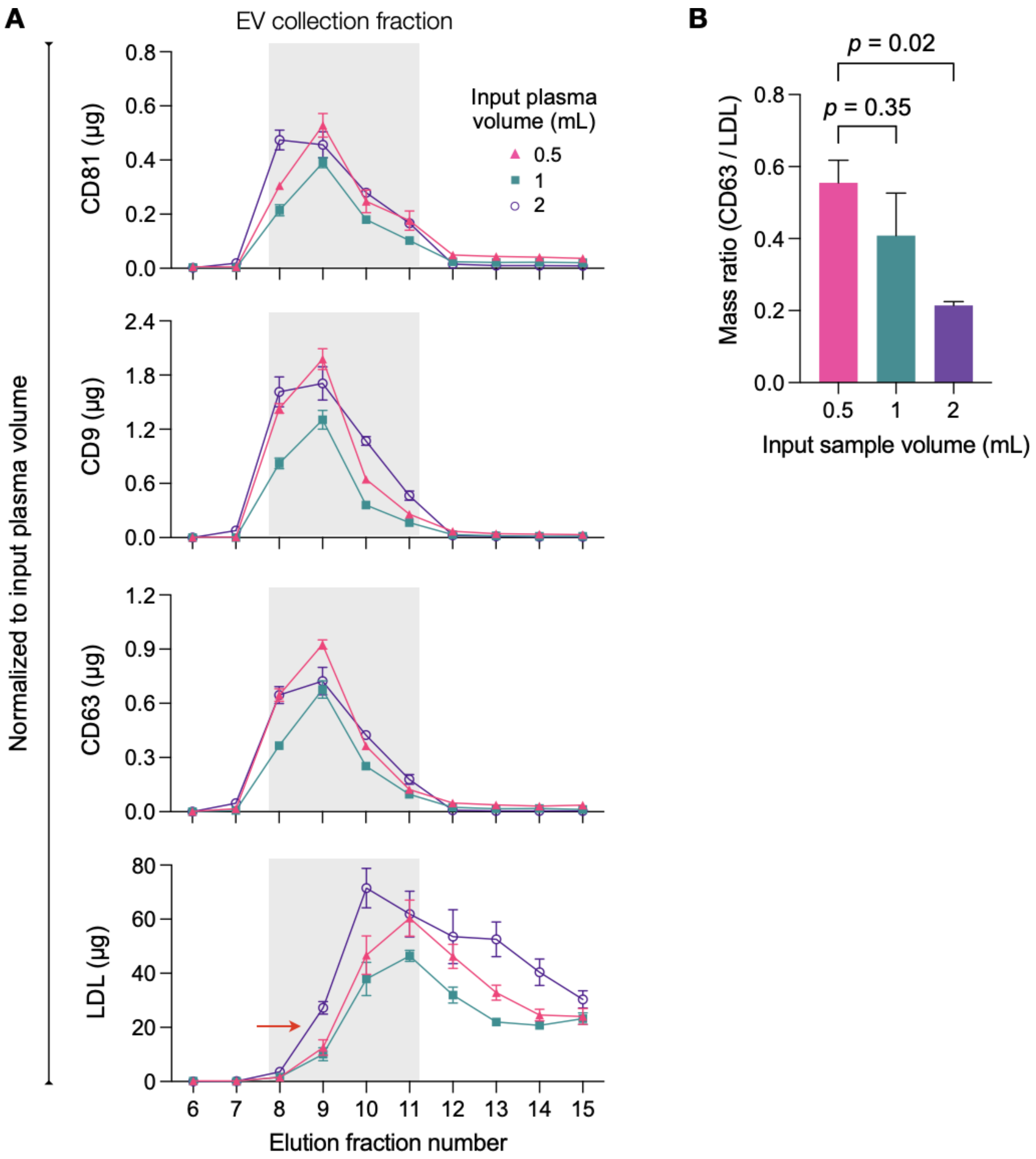

Figure S8. Processing of different volumes of plasma. (A) Human plasma samples (0.5, 1, and 2 mL) spiked with cancer EVs (CaOV3) were processed by eDMC, and eluate sample fractions were analyzed for tetraspanin markers (CD9, CD63, CD81) for EVs and ApoB for LDLs. The grey shadow indicates putative EV-elution fractions (fraction number 8 to 11). More LDLs were found in the EV fraction when 2-mL plasma samples were introduced (red arrow). Data are displayed as mean ±s.d from quadruplicates. (B) EV purity was estimated by taking the mass ratio of CD63 to LDL in the EV elution fractions. the purity was statistically identical between 0.5 and 1 mL plasma inputs, but was significantly lower with 2 mL sample input. Data are displayed as mean ±s.d from duplicates.

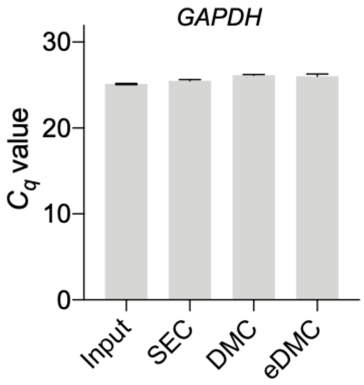

Figure S9. Comparison of *GAPDH* levels in native and filtered plasmas. mRNA was isolated and analyzed through real time PCR. The C_q values were similar in all tested samples. Data from technical triplicates are displayed as mean ± s.d.

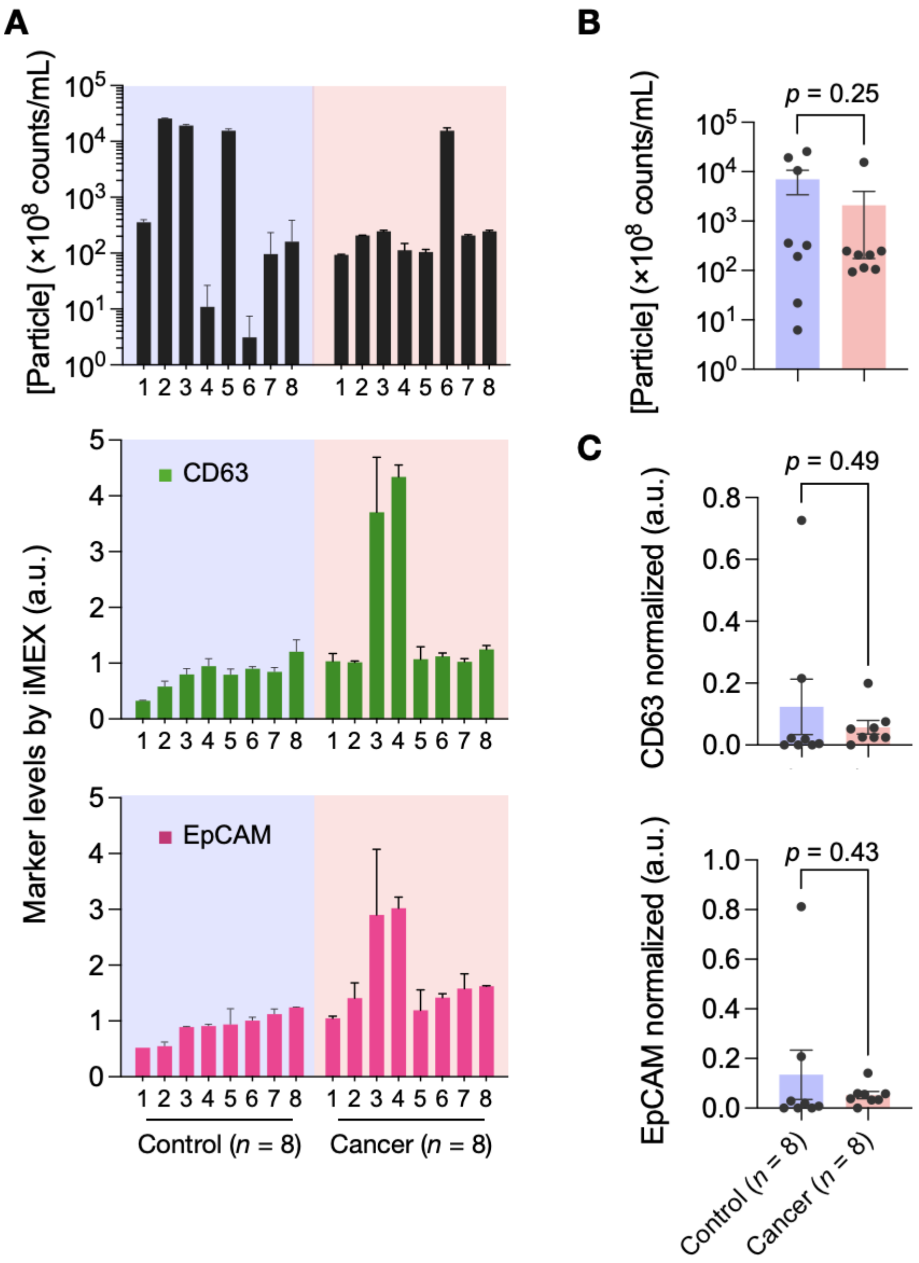

Figure S10. Measurement of particle concentrations in clinical plasma. (A) Plasma samples from healthy donors (n = 8) and cancer patients (n = 8) were processed by eDMC, and the particle concentration in eluate was estimated *via* nanoparticle tracking analysis. CD63 and EpCAM expressions, measured by iMEX, were shown for comparison. **(B)** The particle concentration showed no significant difference between control and cancer cohorts (P = 0.25; two-tailed unpaired t-test). **(C)** Expression levels of CD63 and EpCAM, when normalized against particle concentration, were statistically not different between control and cancer cohorts (P = 0.49 for CD63; P = 0.43 for EpCAM; all two-tailed unpaired t-test).

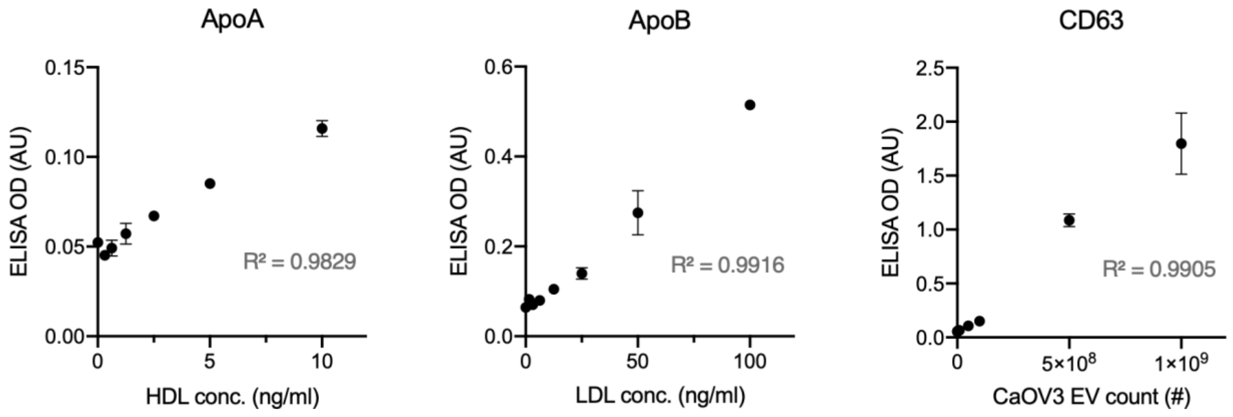

Figure S11. Calibration curves for LPP and EV quantification. Titration curves for ApoA, ApoB, and CD63 were generated via ELISA, and were respectively used to estimate the amount of HDLs, (V)LDLs, and EVs. EVs were obtained from CaOV3 cell line, and their concentrations were measured *via* nanoparticle tracking analysis.

Table S1. List of antibodies used in the current work.

Target	Vendor	Origin	Uses
CD63	Ancell (215-020)	Mouse	ELISA, iMEX, imaging
CD63	Ancell (215-030)	Mouse	ELISA (biotinlyated)
EpCAM	AbCAM (ab187372)	Mouse	iMEX, imaging
ApoAI / ApoB	Cell Biolabs (STA-361)	Mouse	ELISA
Anti-mouse Alexa 647	Thermofisher (31571)	Donkey	imaging

SUPPORTING NOTE – SURFACE CHARGE ESTIMATION

List of symbols

	Description	SI unit	Value
e	electron charge	C	1.6×10^{-19}
k_B	Boltzmann constant	J/K	1.38×10^{-23}
N_a	Avogadro constant	mol^{-1}	6.02×10^{23}
ε	permittivity of water	C/(V•m)	6.99×10^{-10}
T	electrolyte temperature	K	300
σ	surface charge density	C/m^2	
N_1	number density of NH_2 group	m^{-2}	
N_2	number density of COOH group	m^{-2}	
K_1	acid dissociation constant for NH_2 group	M^{-1}	
K_2	acid dissociation constant for COOH group	M^{-1}	
K_m	cation binding constant to COO^-	M^{-1}	0.7 for Na^+
$[H^+]$	proton concentration	M	
$[M^+]$	cation concentration	M	1.37×10^{-4}
c_0	electrolyte concentration	m^{-3}	8.25×10^{22}
ψ_0	surface potential	V	
ζ	zeta potential	V	
z_0	location of the shear plane from the lipid surface	m	2×10^{-10}

1. Ionization Model

We assume that electrical charges on the vesicle surface come from ionization of carboxyl and amino groups. Per framework of the site-dissociation model [3, 4], the charge density is given as

$$\sigma = \frac{eN_1}{1+\frac{K_1}{[H^+]}exp\left(\frac{e\psi_0}{k_BT}\right)} - \frac{eN_2}{1+\left(\frac{[H^+]}{K_2}+K_m[M^+]\right)exp\left(-\frac{e\psi_0}{k_BT}\right)}. \qquad (1)$$

The second term of Eq. (1) includes the charge reduction due to the binding of cations (in electrolyte) to the ionized carboxyl group [4]. From our experiment condition, we used the following values: $pK_1 = -\log_{10}K_1 = 9.1$ (for NH_2), $pK_2 = -\log_{10}K_2 = 3.2$ (for COOH), $[H^+] = 10^{-pH}$. The electrolyte had 0.137 mM NaCl, which gives $[M^+] = [Na^+] = 1.37 \times 10^{-4}$ M. We used $K_m = 0.7\ M^{-1}$ for Na^+ binding to carboxyl group [5].

2. Surface potential

The surface potential in solution is related to a smear charge density through the Gouy-Chapman formula [6],

$$\sigma = \sqrt{8c_0\epsilon k_BT}sinh\left(\frac{e\psi_0}{2k_BT}\right). \qquad (2)$$

The electrolyte (NaCl) concentration c_0 was converted from molarity $[M^+]$ as $c_0 = [M^+] \times N_a \times 10^3$ (m^{-3}) = 8.25×10^{22} (m^{-3}).

To estimate the zeta potential at the distance z_0 from the charged surface, we then used the solution to Poisson-Boltzmann equation,

$$tanh\left(\frac{e\zeta}{4k_BT}\right) = tanh\left(\frac{e\psi_0}{4k_BT}\right) exp\left(-\sqrt{\frac{2e^2c_0}{\epsilon k_BT}} z_0\right). \quad (3)$$

We set z_0 = 0.2 nm for phospholipids [5].

3. Combining all together

Our goal was to estimate N_1 *and* N_2 from measured ζ values. We first used Eq. (3) to estimate ψ_0 from ζ and replaced ψ_0 in Eq. (2) with the calculated value to estimate σ. The obtained ψ_0 and σ were then used in Eq. (1), setting a linear relation between N_1 and N_2. Repeating this process for ζ values measured at different pH, we obtained a set of equations for N_1 and N_2. We then performed the least-square fit to find the optimal N_1 and N_2 values that satisfied the equations.

Supplementary References

1. Sparks DL, Phillips MC. Quantitative measurement of lipoprotein surface charge by agarose gel electrophoresis. J Lipid Res. 1992; 33: 123-130.
2. Hosseini II, Liu Z, Capaldi X, AbdelFatah T, Montermini L, Rak J, Reisner W, Mahshid S. Nanofluidics for Simultaneous Size and Charge Profiling of Extracellular Vesicles. Nano Lett. 2021; 21: 4895-4902.
3. Rendall HM, Smith AL. Surface and Electrokinetic Potentials of Interfaces Containing Two Types of Ionising Group. J Chem Soc, Faraday Trans. 1978; 74: 1179.
4. Tsui FC, Sundberg SA, Hubbell WL. Distribution of Charge on Photoreceptor Disc Membranes and Implications for Charged Lipid Asymmetry. Biophys J. 1990; 57: 85-97.
5. Eisenberg M, Gresalfi T, Riccio T, McLaughlin S. Adsorption of Monovalent Cations to Bilayer Membranes Containing Negative Phospholipids. Biochemistry. 1979; 18: 5213-5223.
6. Lyklema J. Surface Charges and Electrokinetic Charges: Distinctions and Juxtapositionings. Colloids Surf. A. 2011; 376: 2-8.
